# Comparing α-Quartz-Induced Cytotoxicity and Interleukin-8 Release in Pulmonary Mono- and Co-Cultures Exposed under Submerged and Air-Liquid Interface Conditions

**DOI:** 10.3390/ijms23126412

**Published:** 2022-06-08

**Authors:** Alexandra Friesen, Susanne Fritsch-Decker, Matthias Hufnagel, Sonja Mülhopt, Dieter Stapf, Andrea Hartwig, Carsten Weiss

**Affiliations:** 1 Department of Food Chemistry and Toxicology, Karlsruhe Institute of Technology (KIT), Institute of Applied Biosciences, 76131 Karlsruhe, Germany; alexandra.friesen@kit.edu (A.F.); matthias.hufnagel@gmail.com (M.H.); 2 Karlsruhe Institute of Technology (KIT), Institute of Biological and Chemical Systems, Biological Information Processing, 76344 Eggenstein-Leopoldshafen, Germany; susanne.fritsch-decker@kit.edu; 3 Karlsruhe Institute of Technology (KIT), Institute for Technical Chemistry, 76344 Eggenstein-Leopoldshafen, Germany; sonja.muelhopt@kit.edu (S.M.); dieter.stapf@kit.edu (D.S.)

**Keywords:** quartz, pulmonary toxicity, air-liquid interface, co-culture, cytotoxicity, inflammation, A549, THP-1

## Abstract

The occupational exposure to particles such as crystalline quartz and its impact on the respiratory tract have been studied extensively in recent years. For hazard assessment, the development of physiologically more relevant in-vitro models, i.e., air-liquid interface (ALI) cell cultures, has greatly progressed. Within this study, pulmonary culture models employing A549 and differentiated THP-1 cells as mono-and co-cultures were investigated. The different cultures were exposed to α-quartz particles (Min-U-Sil5) with doses ranging from 15 to 66 µg/cm^2^ under submerged and ALI conditions and cytotoxicity as well as cytokine release were analyzed. No cytotoxicity was observed after ALI exposure. Contrarily, Min-U-Sil5 was cytotoxic at the highest dose in both submerged mono- and co-cultures. A concentration-dependent release of interleukin-8 was shown for both exposure types, which was overall stronger in co-cultures. Our findings showed considerable differences in the toxicological responses between ALI and submerged exposure and between mono- and co-cultures. A substantial influence of the presence or absence of serum in cell culture media was noted as well. Within this study, the submerged culture was revealed to be more sensitive. This shows the importance of considering different culture and exposure models and highlights the relevance of communication between different cell types for toxicological investigations.

## 1. Introduction

The human lung is exposed to various substances on a daily basis. Among the numerous chemicals, fibers, and particles known to induce adverse outcomes by inhalation, α-quartz particles remain one of the best studied and most relevant. Especially occupational exposure during processes such as sandblasting, mining, drilling, or manufacturing is associated with a high risk of pulmonary pathologies such as lung cancer, tuberculosis, chronic obstructive pulmonary disease, or silicosis [1,2,3]. Moreover, quartz was classified as carcinogenic in humans (Group 1) by the International Agency for Research on Cancer (IARC) in 1997 [4].

Inflammation and cell injury appear to be early markers for the onset of various lung pathologies, especially silicosis [5,6]. The release of cytokines and chemokines, the infiltration of inflammatory cells, and the development of fibrotic changes as the driving force of pulmonary disease upon quartz exposure in vivo have been covered extensively in the literature [7,8,9,10]. Similar conclusions were drawn from several in vitro investigations assessing cytotoxicity, DNA damage, and inflammation in macrophages [11,12,13,14], epithelial cells [15,16,17], and pulmonary fibroblasts [18,19]. The biological activity of quartz is generally attributed to its surface reactivity, which presents highly reactive silanol groups [20] and contributes to the formation of free radicals and reactive oxygen species [10,19]. The radicals can in turn interact with various cellular components, such as cellular membranes [12] and DNA [12,15]. Furthermore, radical formation and endocytosis of quartz particles promotes inflammatory processes [11] and causes various other effects, e.g., an inflammation-driven secondary genotoxicity [21].

Nevertheless, it is still difficult to estimate the full toxicological potential of a material exclusively from in vitro studies. Both cells and particles often exhibit behavior very different from that under actual physiological conditions. Various cell types are involved in the progression of pulmonary disease caused by quartz particles, especially macrophages, granulocytes, epithelial cells, and fibroblasts [20]. Recently, the development of more advanced in vitro models has been moved forward to represent conditions in the lung more closely. Furthermore, it is imperative to reduce the amount of animal testing required for in vivo investigations in the context of the 3Rs (replace, reduce, refine) [22]. In the context of this initiative, the consideration of cell–cell interactions by employing co- or triple-culture models moved into the spotlight. These models utilize different cell types and analyze interactions by culturing the cells in contact or no-contact scenarios [23]. While immortalized cell lines such as alveolar epithelial A549 cells, bronchial epithelial BEAS-2B cells, or Calu-3 cells can be employed for co-culture studies, even more advanced models composed of stem-cell derived organoids or organ-on-a-chip models are possible [23,24,25]. A further path to physiologically relevant in vitro models is the development of cell models grown at an air–liquid interface (ALI), with cells being exposed to the ambient air from one side. This way, there is the possibility to expose cells at the ALI to various toxicants via aerosols [26,27]. Another point of consideration is the composition of media used for the respective models, as the presence of fetal bovine serum (FBS) is known to alter particle properties and thus influence particle toxicity [28,29,30,31].

3D cultures comprised of the alveolar cell line A549, macrophage-like differentiated THP-1 cells, and endothelial cell lines Ea.hy926 or HIVE-26 have been investigated with regard to the toxicity of α-quartz Min-U-Sil5 [32,33]. A conditioned-media approach with rat epithelial cells and macrophages has been reported as well [34]. Furthermore, one ALI model composed of A549 cells, THP-1 cells, and the fibroblast cell line MRC-5 was exposed to Min-U-Sil5, while other studies utilized ALI exposure of mono-cultured epithelial cells [35]. Thus, the use of co-culture and ALI models is still not common but an emerging approach in inhalation toxicology. Specifically, the differential response of mono- versus co-cultures exposed to particulates at the ALI is rarely compared. Hence, not only the contribution of individual cell types but also the interactions between cell types to generate adverse reactions remains largely unexplored. Similarly, there is only a limited number of studies which perform a direct comparison of epithelial cell mono-cultures and epithelial cell/macrophage co-cultures exposed at the ALI or under conventional submerged conditions [36]. However, such comparative analysis is instrumental to further improve and establish the most sensitive models for hazard assessment and to enable mechanistic studies. It is the overall objective to unravel the distinct role of particular cell types in the various adverse reactions to noxious agents.

For that reason, we utilized A549 cells and differentiated THP-1 cells (dTHP-1) for exposure to the well-studied quartz Min-U-Sil5 and assessed cytotoxicity, proliferation, and cytokine release as common endpoints of toxicological testing. The endpoints of cytotoxicity and inflammation were assessed in mono- and co-cultures under submerged and ALI conditions to perform a comprehensive comparison of different types of cellular models. Moreover, we addressed the impact of serum in the exposure medium on the response of submerged cultures treated with quartz, as in the absence of serum in vitro tests seem to more reliably predict toxicity of particles observed in short term inhalation studies [14]. It was our aim to compare the different cell culture models and routes of exposure to ultimately improve and define more sensitive test systems to investigate particle toxicity.

## 2. Results

Firstly, the cytotoxicity of the α-quartz Min-U-Sil5 was determined by exposing mono-cultures of A549 cells and differentiated THP-1 cells (dTHP-1) and measuring the metabolic activity by AlamarBlue reduction as well as cell death by automated high-throughput microscopy (AHM). Next, the cell lines were combined to form a more complex cell culture model. The resulting co-culture was compared to the A549 mono-culture with regard to lactate dehydrogenase (LDH) release after exposure to three doses Min-U-Sil5 (15, 30, and 60 µg/cm^2^). For all submerged experiments, the applied doses were considered to be equal to the final delivered doses because of the fast deposition of quartz particles in suspension, which has already been reported in literature [37]. To ensure highest possible comparability of the data, the metric of particles per surface area (µg/cm^2^) was chosen. This metric reflects the deposited and measured dose in the ALI experiments and can also be directly calculated for submerged exposure. The release of the chemokine interleukin-8 (IL-8) was determined in all three models by performing an ELISA to evaluate a possible proinflammatory response. Finally, ALI models comprised of A549 mono- and A549/dTHP-1 co-cultures were exposed to corresponding doses of quartz. The ALI cultures were evaluated regarding cytotoxicity (LDH release) and IL-8 release and compared to their submerged counterparts.

### 2.1. Physico-Chemical Properties of α-Quartz Min-U-Sil5

Min-U-Sil5 particles were assessed via transmission electron microscopy (TEM) and dynamic light scattering (DLS) after preparation of particle suspension and via TEM after aerosolization in the ALI exposure chamber. The TEM images are displayed in Supplementary Figure S1, and the DLS measurement in Supplementary Figure S2. Key properties of the assessed particles are depicted in Table 1. The hydrodynamic diameter of the quartz particles ranged from 0.5 to 2 µm with a peak at 0.95 µm, which is in line with published literature [38]. Moreover, the measured polydispersity index (PDI) amounted to 0.462, which indicates that the suspension was polydisperse. The broad size distribution was especially apparent in the TEM images after aerosolization with the Vitrocell Cloud.

### 2.2. Differentiated THP-1 Macrophage-Like Cells Are More Sensitive towards Min-U-Sil5 Than A549 Epithelial Cells as Evidenced by Reduced Metabolic Activity and Enhanced Cell Death

**Figure 1:**
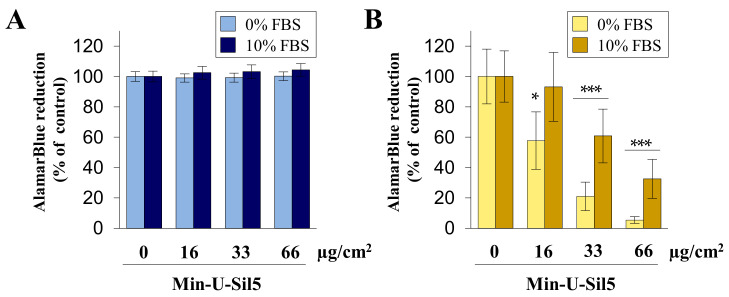
Metabolic activity of A549 (**A**) and dTHP-1 (**B**) cells after submerged exposure to Min-U-Sil5. A549 cells and dTHP-1 cells were treated with 0, 16, 33, or 66 µg/cm^2^ Min-U-Sil5 (0, 50, 100, or 200 µg/mL) either with or without FBS for 24 h. The cells were analyzed for AlamarBlue reduction which is shown relative to the untreated control (100%). The means ± SD of three independent experiments performed in duplicates are displayed. Statistical analysis was performed to assess differences between the cells cultured in media with and without FBS using student’s *t*-test: * (*p* ≤ 0.05), *** (*p* ≤ 0.001).

To assess the toxicity of Min-U-Sil5 particles, A549 and dTHP-1 cells cultured separately under submerged conditions in the presence or absence of FBS were exposed to three doses of particles. The results of the AlamarBlue assay are displayed in Figure 1.

No differences between the negative control and exposed cells were observed in the case of A549 cells, both in the absence and presence of FBS. In contrast, dTHP-1 cells displayed a concentration-dependent decrease in metabolic activity. dTHP-1 cells cultured without FBS were very vulnerable towards Min-U-Sil5, with significant differences to cells cultured with FBS at all concentrations.

**Figure 2:**
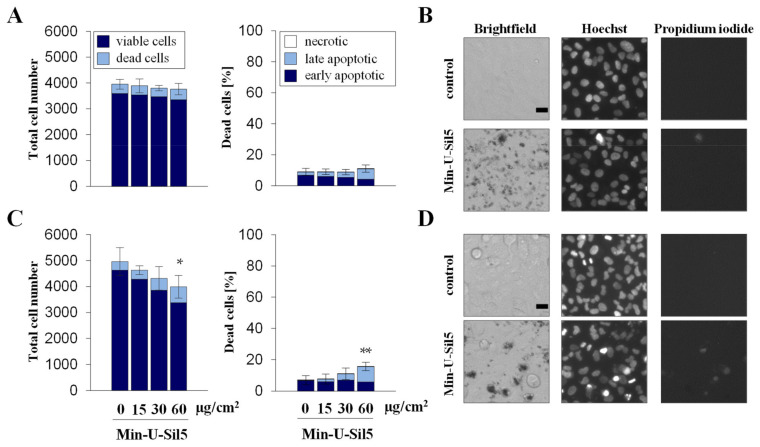
Proliferation and cell death of A549 cells after submerged exposure to Min-U-Sil5 in the absence (**A**,**B**) or presence of FBS (**C**,**D**). A549 cells were treated with 0, 15, 30, and 60 µg/cm^2^ Min-U-Sil5 (0, 50, 100, and 200 µg/mL) for 24 h. The cells were stained with Hoechst and PI and analyzed by automated high-throughput microscopy (AHM). Shown is the total cell number divided into living and dead cells and the percentage of dead cells (**A**,**C**) relative to the total cell number divided into apoptotic (condensed chromatin), late apoptotic (condensed chromatin + PI-positive), and necrotic cells (PI-positive). The means ± SD of one representative experiment out of two each performed in quadruplicates are displayed. Representative images of cells exposed to 200 µg/mL Min-U-Sil5 are shown (**B**,**D**). Statistical analysis was performed to assess differences between treated and untreated cells using student’s *t*-test: * (*p* ≤ 0.05), ** (*p* ≤ 0.01). Scale bar: 20 µm.

**Figure 3:**
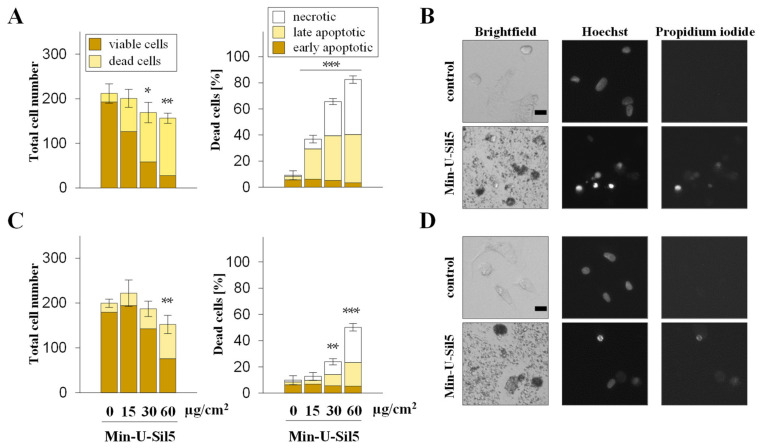
Proliferation and cell death of dTHP-1 cells after submerged exposure to Min-U-Sil5 in the absence (**A**,**B**) or presence of FBS (**C**,**D**). dTHP-1 cells were treated with 0, 15, 30, or 60 µg/cm^2^ Min-U-Sil5 (0, 50, 100, or 200 µg/mL) for 24 h. The cells were stained with Hoechst and PI and analyzed by AHM. Shown is the total cell number divided into living and dead cells and the percentage of dead cells (**A**,**C**) relative to the total cell number divided into apoptotic (condensed chromatin), late apoptotic (condensed chromatin + PI-positive), and necrotic cells (PI-positive). The means ± SD of one representative experiment out of two each performed in quadruplicates are displayed. Representative images of cells exposed to 200 µg/mL Min-U-Sil5 are shown (**B**,**D**). Statistical analysis was performed to assess differences between treated and untreated cells using student’s *t*-test: * (*p* ≤ 0.05), ** (*p* ≤ 0.01), *** (*p* ≤ 0.001). Scale bar: 20 µm.

Quartz exposed A549 and dTHP-1 cells were further assessed via automated high-throughput microscopy (AHM) to investigate the impact on proliferation and cell death. The number of viable and dead cells as well as the percentage of necrotic and apoptotic cells are displayed in Figure 2 (A549) and Figure 3 (dTHP-1) with representative images shown to illustrate the different cellular phenotypes.

A549 cells only showed an increase in dead cells in the presence of FBS at the highest dose of 60 µg/cm^2^, the major part consisting of late-apoptotic but no necrotic cells. Similar to the differences observed above for the two cell types concerning metabolic activity, dTHP-1 cells also appeared more sensitive towards the particles in the AHM assay, especially in the absence of FBS. At the highest dose of 60 µg/cm^2^ most cells cultured without FBS were not viable, whereas half of the cells cultured in the presence of FBS were still alive. In both cases, large proportions of the dead cells were in early or late apoptotic states, with some necrotic cells present as well. The low cell density of dTHP-1 cells depicted in the microscopic images was due to the low number of cells seeded. dTHP-1 cells were seeded deliberately at low numbers (ten times lower than in experiments with A549 mono-cultures) to achieve the same density as applied for A549/dTHP-1 co-cultures. This way, the differential response of A549 and dTHP-1 cells in either mono- or co-cultures can be compared at the same density.

### 2.3. Co-Cultures of A549 and dTHP-1 Cells Show Similar Sensitivity towards Min-U-Sil5 as the Corresponding A549 Mono-Cultures

**Figure 4:**
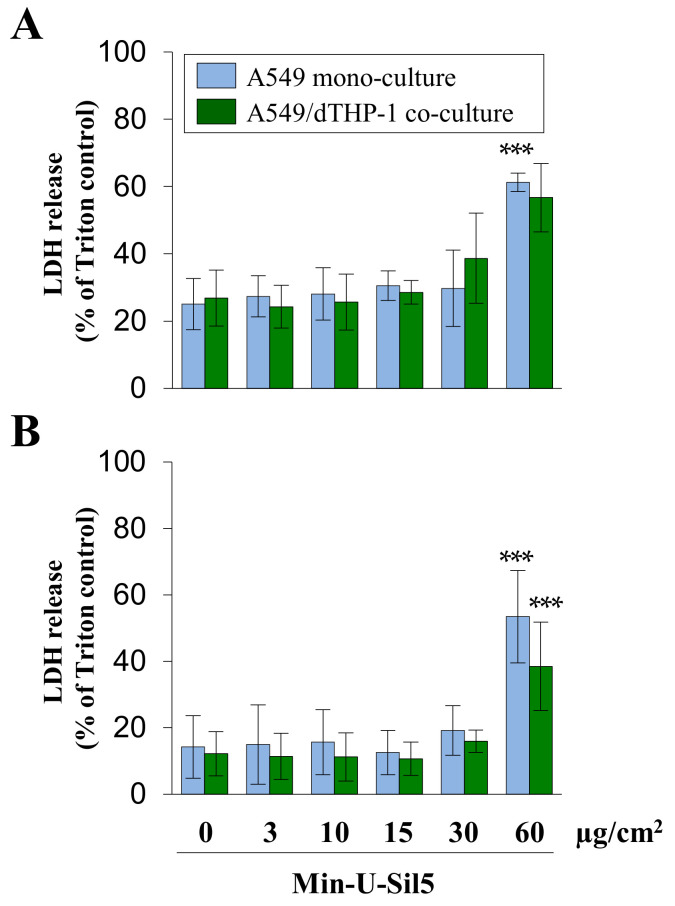
Assessment of cytotoxicity in A549 mono- and A549/dTHP-1 co-cultures after submerged exposure to Min-U-Sil5 in the absence (**A**) or presence of FBS (**B**). A549 cells were seeded with a density of 2 × 10^5^/cm^2^. After 4 h, dTHP-1 cells (2.95 × 10^4^/cm^2^) were seeded on top of the A549 cells for co-culture treatment. On the next day, mono- and co-culture were treated with 3, 10, 15, 30, or 60 µg/cm^2^ (3, 9, 30, 45, 90, or 180 µg/mL) Min-U-Sil5 for 24 h. The supernatants were analyzed for LDH release. LDH release is depicted relative to the Triton control (100%). The means ± SD of at least three independent experiments performed in duplicates are displayed. Statistical analysis was performed to assess differences between treated and untreated cells using student’s *t*-test: *** (*p* ≤ 0.001).

To examine whether interactions between cells have an impact on quartz toxicity, A549 and dTHP-1 cells were seeded as co-cultures and exposed to a wider range of doses of Min-U-Sil5. dTHP-1 were seeded on top of the A549 cells to achieve a physiologically relevant ratio of epithelial cells to macrophage-like cells of 10:1 at the time of exposure. The LDH release after 24 h for both the mono- and the co-culture is displayed in Figure 4.

A significant increase of LDH in the surrounding media was only observed at the highest dose for cells both in the presence and absence of FBS. The initial LDH release of untreated cells and cells exposed to low quartz doses was higher in cells cultured without (>20%) than in cells cultured with FBS (<20%), which can be traced back to a higher share of late-apoptotic and necrotic cells. This shows that even untreated cells are generally more sensitive in the absence of FBS. Differences between mono- and co-cultures were minor, with the mono-culture showing slightly higher LDH releases at the highest quartz dose.

### 2.4. A549/dTHP-1 Co-Cultures Produce Far More IL-8 Compared to dTHP-1 or A549 Mono-Cultures upon Exposure to Min-U-Sil5

**Figure 5:**
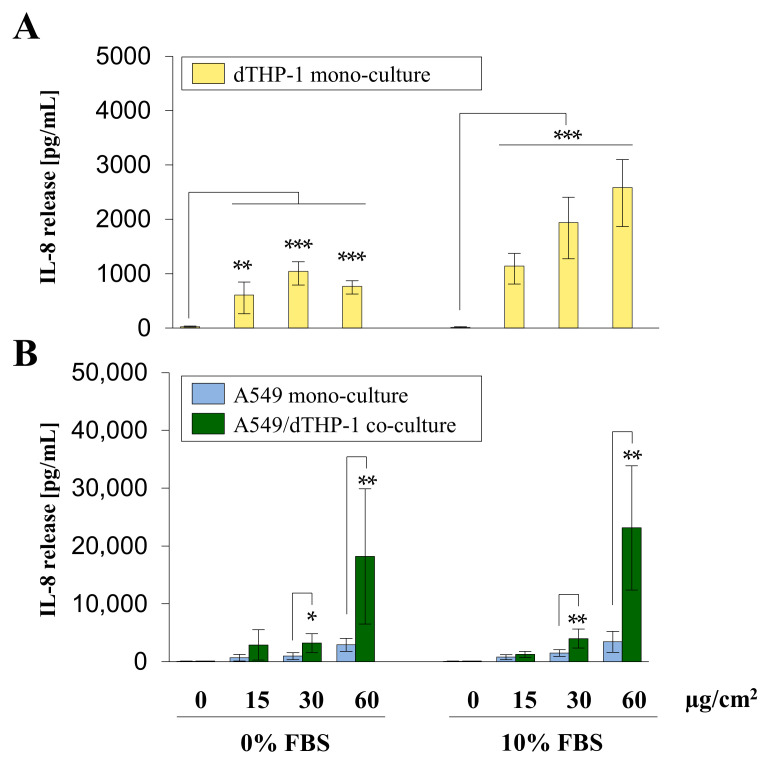
Interleukin-8 (IL-8) release from A549 and dTHP-1 mono-cultures as well as A549/dTHP-1 co-cultures after submerged exposure to Min-U-Sil5. A549 and dTHP-1 cells were seeded as monocultures at a density of 2 × 10^5^/cm^2^ and 2.95 × 10^4^/cm^2^, respectively (**A**,**B**). A549 cells were seeded with a density of 2 × 10^5^/cm^2^. After 4 h dTHP-1 cells (2.95 × 10^4^/cm^2^) were seeded on top of the A549 cells for co-culture treatment (**B**). The next day, mono- and co-cultures were treated with 45, 90, or 180 µg/mL (15, 30, or 60 µg/cm^2^) Min-U-Sil5 either in the presence or absence of FBS for 24 h. The supernatants were analyzed for IL-8 release. The means ± SD of three independent experiments performed in duplicates are displayed. Statistical analysis was performed to assess differences between untreated and treated cells (dTHP-1) and differences between mono- and co-cultures (A549, A549/dTHP-1) using student’s *t*-test: * (*p* ≤ 0.05), ** (*p* ≤ 0.01), *** (*p* ≤ 0.001).

To assess the proinflammatory response of the three models to Min-U-Sil5, interleukin-8 (IL-8) release was investigated by enzyme-linked immunosorbent assay (ELISA). The results are depicted in Figure 5.

dTHP-1 mono-culture showed a significant concentration-dependent increase in IL-8 release in the presence of FBS, whereas in the absence of FBS a peak was reached at the dose of 30 µg/cm^2^ with IL-8 release decreasing at the highest concentration. A549 mono- and A549/dTHP-1 co-cultures exhibited a concentration-dependent increase in IL-8, with minor differences between cells cultured in the absence or presence of FBS. The co-culture released significantly more IL-8 in comparison to both mono-cultures at the middle and highest doses. The concentration range of IL-8 in the co-culture exposed to the highest dose of quartz reached 23 ng/mL IL-8. In comparison, dTHP-1 and A549 mono-cultures showed a maximum release of 2.6 and 3.4 ng/mL, respectively.

### 2.5. Mono- and Co-Cultures Exposed to Min-U-Sil5 at an Air-Liquid Interface Are Less Sensitive Than Their Submerged Counterparts

**Figure 6:**
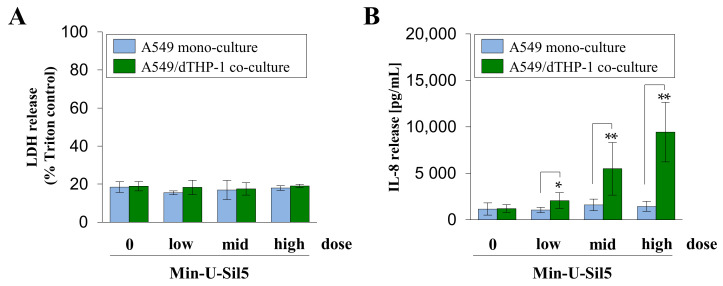
Assessment of cytotoxicity (**A**) and IL-8 release (**B**) from A549 mono- and A549/dTHP-1 co-cultures after air–liquid interface (ALI) exposure to Min-U-Sil5. A549 cells were seeded in trans-well inserts with a density of 2 × 10^5^/cm^2^, followed by dTHP-1 cells in a density of 3 × 10^4^/cm^2^ after 4 h for co-culture treatment. Afterwards, the medium in the apical compartment was removed to establish ALI conditions and the cells were exposed to 15.8 ± 0.1, 29.1 ± 2.1 and 60.9 ± 2.1 µg/cm^2^ (mono-culture) or 16.4 ± 0.5, 31.0 ± 0.6 and 58.1 ± 3.9 µg/cm^2^ Min-U-Sil5 (co-culture) in the Vitrocell Cloud, followed by 24 h post-incubation. Supernatants were analyzed for LDH and IL-8 release. The means ± SD of three independent experiments performed in duplicates are displayed. Statistical analysis was performed to assess differences between the mono- and co-cultures using student’s *t*-test: * (*p* ≤ 0.05), ** (*p* ≤ 0.01).

To evaluate more physiologically relevant conditions, A549 mono- and A549/dTHP-1 co-cultures were exposed to Min-U-Sil5 at the ALI. The deposited doses were chosen corresponding to the doses applied under submerged conditions. dTHP-1 mono-cultures were not suitable for exposure via an ALI since dTHP-1 cells do not form a monolayer and are not viable under ALI conditions. The LDH and IL-8 release of the ALI cultures exposed to quartz are depicted in Figure 6.

Min-U-Sil5 did not induce a cytotoxic response in both mono- and co-cultures, regardless of the applied dose. Furthermore, no significant release of IL-8 was detected in the A549 mono-culture. In contrast, a strong concentration-dependent increase of IL-8 was observed in the co-cultures with a peak concentration of 10 ng/mL IL-8 at the highest dose. These results show that the ALI cultures reacted less sensitively to the quartz particles than their submerged counterparts.

## 3. Discussion

In this work, we applied different pulmonary cell culture and exposure models to investigate adverse effects of quartz using α-quartz Min-U-Sil5. Lung epithelial cells and macrophages were employed as important target cells of particles, and as read-outs with relevance for lung pathology the cytotoxicity and the release of the chemokine IL-8 as a central regulator of neutrophil recruitment were investigated. Firstly, submerged mono-cultures of A549 and differentiated THP-1 (dTHP-1) cells were assessed with regard to metabolic activity, proliferation, and cell death in a classical exposure approach. Next, a submerged A549/dTHP-1 co-culture model was established and evaluated regarding cytotoxicity in comparison to the A549 mono-culture. Finally, corresponding air–liquid interface (ALI) models of the A549 mono- and A549/dTHP-1 co-culture were set up and exposed to quartz-containing aerosols followed by cytotoxicity determination. Furthermore, interleukin-8 (IL-8) release was measured for all models. All submerged exposures were performed both in the presence and the absence of FBS, as previous studies indicated a better prediction of particle toxicity observed in inhalation studies by performing in vitro studies in the absence of serum [14]. The aim of the investigation was to compare the different models with respect to sensitivity to further optimize toxicological testing and to explore the interdependence of cytotoxicity and the proinflammatory response induced by quartz particles.

In case of the submerged A549 mono-culture, no changes in metabolic activity were apparent via the AlamarBlue assay. As analyzed by microscopy, a slight decrease in proliferation as well as a significant increase in late-apoptotic cells was observed at the highest quartz concentration in the presence of FBS. A significant LDH release was detected at the highest quartz dose as well. All these results indicate the onset of a cytotoxic response starting at a dose of 60 µg/cm^2^ Min-U-Sil5. Similar results of a low or absent cytotoxic response in A549 cells have been reported in several other studies for Min-U-Sil5 [39,40] or other quartz particles such as Norquartz [41], DQ12 [9], and NIST1878A [15,42]. In all these cases, cytotoxicity was apparent at a concentration of at least 50 µg/cm^2^, which is in line with our findings. Contrastingly, Freyria et al. reported a concentration-dependent and significant cytotoxicity in A549 cells at 20 µg/cm^2^ Min-U-Sil5 [43]. Similar results were observed by Williams et al. with A549 cells showing a slight cytotoxicity to quartz at a low dose of 15 µg/cm^2^ [16]. Regarding apoptosis and cell death, the aforementioned study by Freyria et al. noted a significant increase in apoptotic and necrotic cells starting at a dose of 20 µg/cm^2^. Again, most other studies are in contrast to these findings, with no evident apoptosis or necrosis in A549 cells after exposure to Min-U-Sil5 [40], and only a slight induction of apoptosis after exposure to NIST1878A quartz [42] or Norwegian quartz particles [44], in agreement with our results. A potential explanation for the cytotoxicity found at somewhat lower doses [16,43] could be the different preparation of particle suspensions and application of different cell densities during exposure.

A slight concentration-dependent release of the chemokine IL-8 was observed in the A549 mono-culture, though with a maximum of about 3.4 ng/mL at the highest dose, which is roughly 7–9-fold lower than the release in the corresponding co-cultures. For cells cultured in the absence or presence of FBS, no differences could be found, which suggests that IL-8 release is not affected by the presence of FBS. The induction of IL-8 in A549 cells after exposure with similar doses of quartz has already been reported for Min-U-Sil5 [45,46], Norquartz [41], and NIST1878B quartz [16].

dTHP-1 cells generally reacted more sensitive to Min-U-Sil5. A strong concentration-dependent decrease in metabolic activity was observed, starting already at the lowest quartz dose. This effect was amplified in the absence of FBS. Assessment of cell death and proliferation showed corresponding results: a concentration-dependent increase in necrotic and late-apoptotic cells was observed starting at the middle dose of 30 µg/cm^2^ in the presence of FBS and at the lowest dose of 15 µg/cm^2^ in the absence of FBS. In comparison, A549 cells showed much lower cytotoxicity evidenced by moderate apoptotic cell death but no necrosis. The higher susceptibility of dTHP-1 cells can be explained by the higher rate of internalization of the particles through phagocytosis, which has already been reported in other studies for Min-U-Sil5 and DQ12 [47,48,49]. Furthermore, quartz particles are known to induce lysosomal rupture in macrophages, which leads to the repeated release of particles and lysosomal enzymes and thus a higher cytotoxic response [50]. For amorphous silica particles, coating of the reactive surface by serum suppresses lysosomal membrane damage and consequently cell death [51]. Reactive silanol groups at the surface of crystalline but also amorphous silica seem to be responsible for perturbing the integrity of cellular membranes and are presumed molecular initiating events in the adverse response [20]. Corresponding to our findings, a concentration-dependent cytotoxicity of quartz has been reported in the literature. Some studies demonstrated cytotoxicity at similar concentrations as applied in our work [49,52], whereas in other publications a lower cytotoxicity was evident [53,54,55]. The variations between the different studies could be the result of different methods investigating cytotoxicity, experimental procedures during cell culture and exposure, particle preparation, and cell density. Especially the study by Leinardi et al. showed results similar to the ones within this study, as a comparison between cells cultured in the presence or absence of FBS was performed as well. Their study categorized Min-U-Sil5 particles to be highly cytotoxic to macrophages, even more so in the absence of FBS, and to induce an increase in necrotic cells, which mirrors the results in this work [49]. The mitigating effect of FBS on quartz toxicity has been shown as well in another study with human and rat macrophages [56]. Moreover, apoptotic effects of quartz including activation of caspases have been reported for human alveolar macrophages [48] and murine macrophages [57,58].

The proinflammatory response of dTHP-1 cells to Min-U-Sil5 was assessed by IL-8 ELISA. In contrast to the cytotoxicity results, IL-8 release was stronger in the presence of FBS. Additionally, a decrease of IL-8 was observed in the absence of FBS at the highest concentration. This can be explained by the higher cytotoxicity when cells were cultured without FBS. Especially at the highest dose, production of IL-8 was impaired probably as a result of increased cell death. A similar effect was noted in a study with amorphous silica particles [59] and by Xu et al., even at comparable quartz concentrations [55]. The range of IL-8 release in this study was similar to the release from A549 cells, although it has to be noted that dTHP-1 cells were seeded at a 7-fold lower density. The reduced number of dTHP-1 cells and the earlier onset of cytotoxicity compared with A549 cells explains the similar release, although macrophages are naturally more immunocompetent [60]. IL-8 release upon Min-U-Sil5 exposure starting at similar concentrations as in this study, but to a higher degree, was spotted in a work by Grytting et al. [52].

The A549/dTHP-1 co-culture showed a similar behavior with regard to cytotoxicity as the A549 mono-culture. A significant LDH release was only observed at the highest dose of 60 µg/cm^2^. In the presence of FBS, the co-culture showed a slightly lower but statistically not significantly different LDH release than the mono-culture. Since this dose was shown to be highly cytotoxic for dTHP-1 cells grown as mono-culture, whereas no additional LDH release was noted in the co-culture, the absence of a further increase of LDH in the co-culture is most likely explained by the lower number of dTHP-1 cells used in the co-culture versus the mono-culture. Comparable patterns of less cytotoxicity in co-cultures as compared to mono-cultures were reported in A549 and A549/dTHP-1 models exposed to Min-U-Sil5 [52], PM_2.5_ [61], and hAELVI epithelial cells co-cultured with THP-1 cells upon exposure with silver and starch particles [62]. Similar co- or 3D culture models have been assessed concerning Min-U-Sil5 toxicity, albeit the results differ drastically: in a work examining Min-U-Sil5 toxicity in a 3D culture model composed of A549, dTHP-1, and endothelial Ea.hy926 cells no cytotoxic effect was detected, even at a concentration as high as 192 µg/cm^2^ [33]. Similar results were reported in experiments with co-cultures comprised of primary rat macrophages and alveolar cells [63]. In contrast, an A549/THP-1 co-culture model showed a strong cytotoxic response at a concentration of 160 µg/cm^2^, although in this particular model THP-1 cells were applied as undifferentiated monocytes [32]. These reports highlight the impact of 3D vs. 2D cultivation as well as the influence of differences in cell type and co-culture composition on the final dose–response and severity of the cytotoxic effects of quartz.

The submerged A549/dTHP-1 co-culture displayed the highest release of IL-8 in this study, with a peak concentration of around 23 ng/mL and no apparent differences in the presence or absence of FBS. Thus, the co-culture released around ten times more IL-8 than both the A549 and dTHP-1 mono-cultures, which suggests that cell-to-cell communication between the two cell types critically determines the magnitude of the response. Presumably, an initial inflammatory response with various secreted cytokines, such as TNF alpha, takes place in the macrophages, which in turn sensitize the epithelial cells to release more IL-8. In fact, this has already been demonstrated for a BEAS-2B/dTHP-1 co-culture exposed to fly ash [64], a A549/dTHP-1 co-culture exposed to DQ12 quartz and other particles [65], as well as a no-contact A549/dTHP-1 co-culture exposed to Min-U-Sil5 [45].

Finally, ALI models based on the A549 mono- and A549/dTHP-1 co-culture were assessed with regard to the same endpoints. In contrast to their submerged counterparts, there was no cytotoxic response triggered by Min-U-Sil5 at all doses. Additionally, the ALI cultures released less IL-8, with a peak concentration of 10 ng/mL in the co-culture. Therefore, the ALI models appeared less sensitive towards quartz particles for both endpoints. Moreover, the proinflammatory response was initiated by quartz, even though no cytotoxicity was triggered, suggesting that both events can be uncoupled and are independent of each other as previously shown also in the case of amorphous silica particles [66]. Studies comparing ALI and submerged exposure are scarce and show contradictory results. While in most studies, ALI grown cells are reported to be the more sensitive model, such as after exposure to engineered nanoparticles [67], PM_2.5_ [68], TiO_2_, or CeO_2_ nanoparticles [69,70,71], some show similar responses, such as after TiO_2_ exposure [72]. Apparently, the agglomeration of particles is also important, as agglomerates of small TiO_2_ particles induce more cytotoxicity at the ALI compared with submerged exposure, whereas agglomerates of larger TiO_2_ particles provoked more DNA damage in submerged versus ALI cultures [73]. One study with silica nanoparticles and A549 cells revealed the submerged exposure model to be more sensitive than the ALI model [74]. The most direct comparison can be performed considering some recent research exposing A549 cells to Min-U-Sil5 at doses up to 300 µg/cm^2^. ALI cultured models appeared less vulnerable to the particles than submerged models, which is in line with our results [75]. In this work, differences between the two exposure types could be in part due to the formation of a different protein corona around the aerosolized particles, as they come into contact with bovine serum albumin (BSA) during particle preparation whereas under submerged conditions the full spectrum of bovine serum proteins is present. Another plausible explanation is the production of surfactant during ALI culture, which has been reported previously for A549 cells and might reduce the toxicity of quartz particles [75,76].

In summary, we were able to document distinct toxicological responses for the different cell culture and exposure models tested. The results generated by exposure of the A549 and dTHP-1 mono-cultures reflect findings from the literature. Exposure of the co-culture demonstrated the importance of considering communication between different cell types resulting in an enhanced proinflammatory response. Finally, application of an air–liquid interface exposure model with more physiological relevance leads to mitigation of both the cytotoxic and inflammatory effects. In the future, these findings should be complemented by the analysis of different cellular models including other established cell lines of different origin such as BEAS-2B and Calu-3 epithelial cells or primary cells.

Intriguingly, the mode of cell death in different target cells, i.e., A549 and dTHP-1 cells, might be of high relevance also in vivo and related to pathological findings. Necrosis, but not apoptosis, potently triggers inflammation in vivo [77]. Therefore, more refined studies should address the individual role of different cell types, including epithelial cells and macrophages, and the molecular pathways of cell death with respect to pathological endpoints such as inflammation upon particle exposure. Moreover, further mechanistical insights into quartz toxicity, by, e.g., employing transcriptomics such as high-throughput RT-qPCR [78] or other assays could be employed to fully uncover the mode of action and toxicity pathways.

## 4. Materials and Methods

### 4.1. Materials

All chemicals, cell culture media, and supplements for cell culture and submerged exposure were purchased from Gibco/Thermo Fisher Scientific GmBH (Dreieich, Germany), and all chemicals for ALI exposure were obtained from Sigma-Aldrich Chemie GmBH (Taufkirchen, Germany) or Carl Roth GmbH (Karlsruhe, Germany), except for fetal bovine serum (FBS), which was obtained from Thermo Fisher Scientific GmBH (Dreieich, Germany). Cell culture consumables such as plates, flasks, and centrifuge tubes were purchased from Sarstedt (Nuembrecht, Germany) and Greiner (Kremsmünster, Germany). Trans-well plates and inserts were bought from Corning (Amsterdam, The Netherlands). Min-U-Sil5 quartz particles were purchased from U.S. Silica (Katy, TX, USA). Endotoxin contamination was measured via LAL Chromogenic Endotoxin Quantitation Kit (ThermoFisher Scientific, Karlsruhe, Germany). The LDH assay kits were acquired from Promega (Madison, WI, USA) and Roche (Mannheim, Germany). Alamar Blue was obtained from AbD Serotec (Purchheim, Germany). IL-8 ELISAs were purchased from Invitrogen (Waltham, MA, USA).

### 4.2. Particle Preparation and Characterization

For submerged exposure, 10 mg/mL quartz suspensions were prepared in sterile water and sonicated using a Branson Sonifier 250 (Brookfield, CT, USA) for 15 s. The particles were assessed by transmission electron microscopy (TEM) after preparation. Particle suspensions were centrifuged at 20,800× *g* for 10 min. The supernatants were tested for endotoxin content according to the instructions of the manufacturer. The endotoxin content was below the lower limit of quantification (>0.1 EU/mg, EU = endotoxin unit).

For ALI exposure, particles were dispersed employing a variation of the latest NANOGENOTOX protocol [79]. Briefly, a defined amount of particles was pre-wetted with 30 µL 97% ethanol before adding 5.970 mL 0.05% bovine serum albumin (BSA) solution. Particle suspensions were sonicated using a Branson Analog Sonifier 450 (Brookfield, CT, USA) for a duration of 13.25 min at 10% amplitude (7197 J). Subsequently, suspensions were aliquoted and frozen at −20 °C. For experiments, suspensions were thawed and sonicated again using a sonication bath for 10 min. Particle properties before and after freezing were monitored by dynamic light scattering (DLS) with the Zetasizer NanoZS (Malvern Panalytical, Herrenberg, Germany). To determine the state of the particles after aerosolization, TEM grids were placed inside the exposure chamber with images of the grids being taken afterwards.

TEM was performed using the UM910 microscope (Zeiss, Oberkochen, Germany).

### 4.3. Cell Culture

The alveolar epithelial cell line A549 (ATCC CCL-185) [80] was kindly provided by Dr. Roel Schins (Leibniz Research Institute for Environmental Medicine, Duesseldorf, Germany). The monocytic cell line THP-1 (ATCC TIB-202) [81] was kindly provided by Dr. Richard Gminski (Albert-Ludwig-University Freiburg, Department of Environmental Health Sciences and Hygiene, Freiburg, Germany). THP-1 cells were differentiated by incubation with 30 ng/mL phorbol 12-myristate 13-acetate (PMA) for five days, followed by an incubation period of three to five days with PMA-free medium to achieve a macrophage-like phenotype (differentiated THP-1: dTHP-1) [82]. Both cell lines were cultured in RPMI-1640 medium, supplemented with 10% fetal bovine serum (FBS), 100 U/mL penicillin and 100 µg/mL streptomycin, and kept at 37 °C in a humidified atmosphere of 5% CO_2_. Only passages 17–55 and 3–22 were used for exposure experiments for A549 and THP-1 cells, respectively.

### 4.4. Particle Exposure

Cells were seeded in 12-, 24-, or 96-well plates for submerged exposure and in 12-well trans-well plates 24 h prior to exposure. The applied models were previously described in a study examining CuO toxicity [83]. Briefly, A549 cells were seeded at a density of 1.96 × 10^5^ cells/cm^2^ to form a tight monolayer after 24 h. For the co-cultures, four to six hours after A549 seeding, dTHP-1 cells were seeded on top at a density of 2.95 × 10^4^ cells/cm^2^. The same density was used for dTHP-1 mono-cultures. The densities for the co-culture were specifically chosen to reproduce physiological conditions in a healthy lung as closely as possible: the ratio of epithelial cells to macrophage-like cells is supposed to amount to 10:1 at the time of exposure [84].

For submerged exposure, Min-U-Sil5 particles were prepared as described and resuspended in full cell culture media or media without FBS.

The procedure for ALI exposure via Vitrocell^®^ Cloud was described previously [85]. Briefly, the Cloud was disinfected with 80% ethanol. One hour before intended exposure, media were removed from the apical compartment of the trans-well inserts to accustom the cells to ALI conditions. Particle suspensions were prepared as described above. The trans-well inserts were transferred to the pre-warmed exposure station and exposed to the particle aerosol for 10 min. The inserts were transferred back to plates with fresh full cell culture media for post-incubation. Deposition of quartz particles was measured by a quartz crystal microbalance (QCM). The deposition was determined by calculation of the means of the last 100 data points.

After a post-incubation time of 24 h, the media were collected to perform cytotoxicity assessment via the lactate dehydrogenase assay. Media were frozen at −20 °C to perform cytokine measurements via IL-8 ELISA at a later time. Cells were detached with 0.25% trypsin solution (A549 mono-cultures) or accutase (co-cultures and dTHP-1 mono-cultures) to determine the cell count. Metabolic activity was measured by the Alamar Blue assay and cell proliferation by automated high-throughput microscopy (AHM).

### 4.5. Cytotoxicity Assessment via Lactate Dehydrogenase (LDH) Assay

The lactate dehydrogenase (LDH) release was assessed with the Cytotoxicity Detection Kit (Sigma-Aldrich, Taufkirchen, Germany) or CytoTox-ONE Homogeneous Membrane Integrity Assay (Promega, Madison, WI, USA) following the manufacturers’ instructions. Absorbance at 490 nm (Cytotoxicity Detection Kit) or fluorescence at 560 nm (exc.) and 590 nm (em.) (CytoTox-ONE) were measured using a plate reader. Positive control cells were lysed with 0.1% Triton X-100 shortly before the end of the post-incubation time and used as a maximum LDH release control.

### 4.6. Assessment of Metabolic Activity by Alamar Blue Assay

To measure the metabolic activity of the lung cell culture, the Alamar Blue Assay was performed as described previously [59]. Briefly, cell culture media were removed after post-incubation and 10% Alamar Blue in Hanks’ balanced salt solution (HBSS) was added, followed by an incubation for 1–2 h. Resorufin fluorescence was measured with a plate reader at 580 nm (exc.) and 620 nm (em.). Fluorescence intensity was normalized to the untreated control cells.

### 4.7. Assessment of Proliferation and Cell Death by Automated High-Throughput Microscopy (AHM)

To assess cell number and different stages of cell death after quartz treatment, automated high-throughput microscopy (AHM) was performed as described previously [86]. Briefly, cells were exposed to quartz particles in 96-well plates for 24 h. After the incubation cells were stained with 0.3 µg/mL Hoechst 33,342 and 0.25 µg/mL propidium iodide (PI) for 30 min. Four pictures per well were taken with the automated fluorescence microscope IX81 (Olympus, Hamburg, Germany) with a ten-fold objective using the bright field, Hoechst, and PI channels. Automated image analysis was performed with the Olympus ScanR analysis software. To obtain the total cell number, Hoechst-stained nuclei were counted. A differentiation between living, early-apoptotic, late-apoptotic, and necrotic cells was possible by comparing Hoechst signal intensities and PI staining.

### 4.8. Cytokine Release via Interleukin-8 Enzyme-Linked Immunosorbent Assay (IL-8 ELISA)

IL-8 release was monitored via IL-8 Human Uncoated ELISA Kit (ThermoFisher Scientific, Karlsruhe, Germany) following the manufacturer’s instructions. Briefly, 96-well plates were coated with a capture antibody, followed by blocking of the plate. Frozen media samples were thawed and diluted to fit into the range of the standard. Standards and samples were added to the 96-well plates and incubated for two hours at room temperature or overnight at 4 °C, followed by incubation with a detection antibody and a conjugate of avidin and horseradish peroxidase. 3,3′,5,5′-Tetramethylbenzidine was added as a substrate followed by the addition of H_2_SO_4_ to stop the reaction. Absorption at 450 nm was measured with a microplate reader.

### 4.9. Statistical Analysis

Statistical analysis was performed with a two-tailed student’s *t*-test to assess the significance of changes between quartz treated and untreated samples, between cells cultured in the presence and absence of FBS and between mono- and co-cultures.

## 5. Conclusions

In the course of this study, we assessed different cell culture models with regard to cytotoxicity and inflammation upon exposure to the α-quartz Min-U-Sil5, starting with a simple submerged A549 model and adding growing layers of complexity by constructing co-cultures with differentiated THP-1 cells and culturing cells at an ALI. The mono-cultures exhibited a low and high cytotoxic response for A549 and dTHP-1 cells, respectively, as well as a similarly ranged release of the inflammatory mediator IL-8. Furthermore, a significant induction of apoptosis and necrosis was observed for dTHP-1 cells. While most of these findings are in line with already published literature, we discovered that co-cultures show drastically enhanced cytokine release while cytotoxicity was similar in mono- and co-cultures. This shows the importance of considering cell-to-cell communication for a more realistic appraisal of pulmonary toxicity of specifically quartz and particles in general. Applying quartz via an ALI system reduced the cytotoxic response even further, while also lowering the proinflammatory response. These results demonstrate that toxicological effects detected by classical submerged exposure might be exaggerated compared with the more physiological exposure at the ALI.

In conclusion, the type of cell culture and exposure model has a great impact on the scope of toxicological responses in-vitro. To further optimize and develop new approach methodologies in the field of particle toxicology, it is important to mimic the actual in vivo situation as closely as possible by establishing more advanced co-culture systems for in vitro testing. More studies are warranted to compare side-by-side noxious effects for different types of particulate matter using both exposure scenarios, i.e., at the ALI and with conventional submerged systems, to finally identify the most sensitive and predictive assays for the prediction of pulmonary toxicity.

## Figures and Tables

**Table 1:** Summary of physico-chemical properties of Min-U-Sil5.

Ø (µm)	PDI	Median Ø (µm)	Purity (%)
0.96 ^1^	0.462 ^1^	1.6 ^2^	99.4 ^2^

^1^ Assessed via dynamic light scattering after preparation of suspension according to the NANOGENOTOX protocol. ^2^ Manufacturer’s information.

## Data Availability

The data presented in this study are available on request from the first (A.F., S.F.-D.) and corresponding author (A.H., C.W.) for researchers of academic institutes who meet the criteria for access to the confidential data.

## Supplementary Materials

The following are available online at https://www.mdpi.com/article/10.3390/ijms23126412/s1.

## Abbreviations

AHM	Automated high-throughput microscopy
ALI	Air-liquid interface
BSA	Bovine serum albumin
DLS	Dynamic light scattering
dTHP-1	differentiated THP-1 cells
ELISA	Enzyme-Linked immunosorbent assay
FBS	Fetal bovine serum
IL-8	Interleukin-8
LDH	Lactate Dehydrogenase
PMA	Phorbol 12-myristate 13-acetate
TEM	transmission electron microscopy
