# Supplementary material for: Comparing α-Quartz-Induced Cytotoxicity and Interleukin-8 Release in Pulmonary Mono- and Co-Cultures Exposed under Submerged and Air-Liquid Interface Conditions
Source: Int J Mol Sci. 2022 Jun 8;23(12):6412. doi: 10.3390/ijms23126412 (PMC9224477; doi:10.3390/ijms23126412)
Supplement: Supplementary file 1 [file ijms-23-06412-s001.zip › ijms-1733926-supplementary.pdf]

Supplementary information to:

Comparing α-quartz-induced cytotoxicity and interleukin-8 release in pulmonary mono- and co-cultures exposed under submerged and air-liquid interface conditions

Alexandra Friesen[1,†], Susanne Fritsch-Decker[2,†], Matthias Hufnagel[1], Sonja Mülhopt[3], Dieter Stapf[3], Andrea Hartwig[1,*] and Carsten Weiss[2,*]

[1] Karlsruhe Institute of Technology (KIT), Institute of Applied Biosciences, Department of Food Chemistry and Toxicology, Karlsruhe Germany
[2] Karlsruhe Institute of Technology (KIT), Institute of Biological and Chemical Systems, Biological Information Processing, Eggenstein-Leopoldshafen, Germany
[3] Karlsruhe Institute of Technology (KIT), Institute for Technical Chemistry, Eggenstein-Leopoldshafen, Germany
* Correspondence: andrea.hartwig@kit.edu, carsten.weiss@kit.edu
† These authors contributed equally to this work.

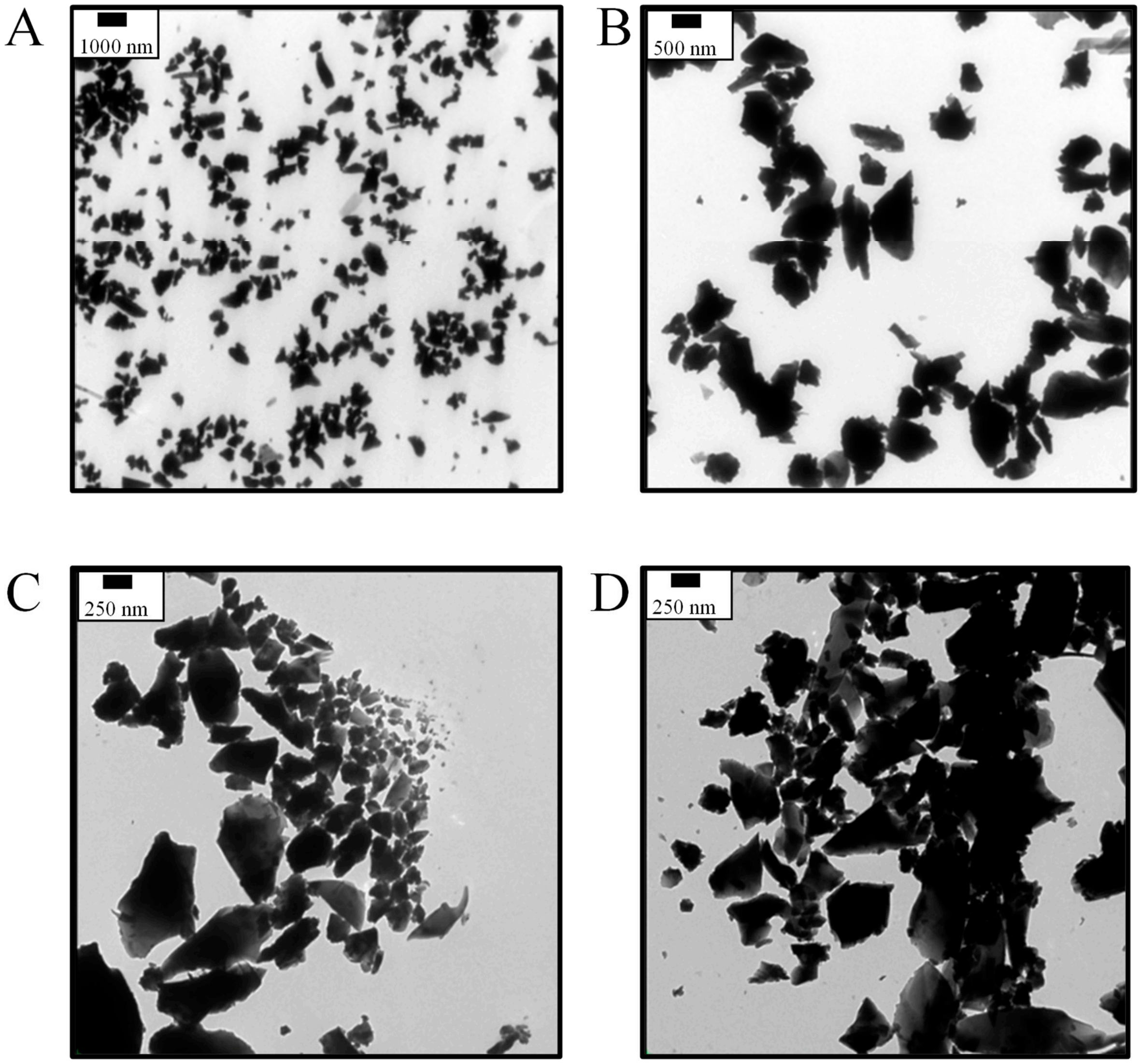

Supplementary Figure S1: Transmission electron microscopy (TEM) of Min-U-Sil5 particles after sonication for submerged experiments (A, B) and after aerosolization with the Vitrocell Cloud (C, D). TEM grids were loaded with a 1 mg/mL suspension of Min-U-Sil5 quartz in water after sonication for 15 seconds (A, B) or by placing them inside the exposure chamber alongside the air-liquid interface experiments at a deposition of 15.22 μg/cm^2 (C) and 58.08 μg/cm^2 (D).

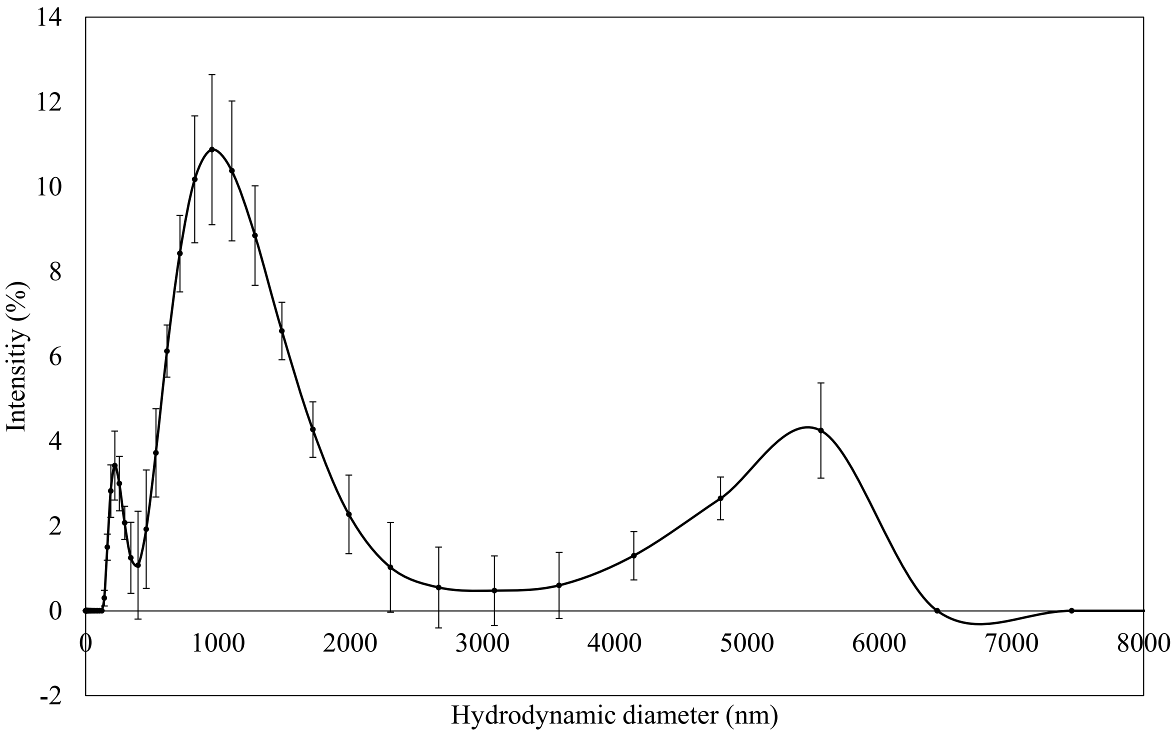

Supplementary Figure S2: Size distribution of Min-U-Sil5 particles measured by dynamic light scattering (DLS). Size distribution of particles measured by DLS at a concentration of 15 mg/mL. The quartz particles were suspended in 0.05 % bovine serum albumin (BSA) solution, sonicated in accordance with the NANOGENOTOX protocol and frozen at -20 °C. Before cell culture experiments and DLS measurements, the suspensions were thawed and re-sonicated in a sonication bath. The results from two independent experiments performed in triplicates are displayed ± SD.
